# RDT- AND MICROSCOPIC-BASED TRENDS OF MALARIA AMONG LOCAL AND MIGRANT POPULATIONS IN THE PROVINCE OF CENTRAL KALIMANTAN, INDONESIA DURING 2017-2020

**DOI:** 10.21010/Ajid.v16i2S.1

**Published:** 2022-08-17

**Authors:** Arwati Heny, Lestarisa Trilianty, Augustina Indria, Rohmah Etik Ainun, Subekti Sri, Keman Soedjajadi, Dachlan Yoes Prijatna

**Affiliations:** 1 *Department of Medical Parasitology, Faculty of Medicine, Universitas Airlangga, Surabaya, Indonesia; 2 Department of Public Health, Faculty of Medicine, Universitas Palangka Raya, Palangka Raya City, Indonesia; 3 Department of Parasitology, Faculty of Medicine, Universitas Palangka Raya, Palangka Raya City, Indonesia; 4 Department of Entomology, Institute of Tropical Diseases, Universitas Airlangga, Surabaya, Indonesia; 5 Department of Marine, Faculty of Fisheris and Marine, Universitas Airlangga, Surabaya, Indonesia; 6 Department of Environmental Health, Faculty of Public Health, Universitas Airlangga, Surabaya, Indonesia

**Keywords:** Malaria, Indonesia, Central Kalimantan Province, Microscopy, RDT

## Abstract

**Background:**

Indonesia has demonstrated a significant progress in malaria elimination. Kapuas and Gunung Mas Districts in Central Kalimantan Province have not been freed from malaria and there is no information of malaria incidences in these areas. Palangka Raya city has been freed from malaria in 2018.

**Materials and Methods:**

The total number of 140 samples consisting of 75 malaria Giemsa-stained blood smears and 65 RDT cartridges from both local and migrant populations. Both males and females aged15 years and above were included and their demographic data were recorded. The malaria trend in these areas was analyzed based on the number of cases, species of *Plasmodium* and the demographic characteristics of the enrolled subjects.

**Results:**

The study findings disclosed a yearly decrement of malaria trend in both local and migrant populations of the studied areas. The highest number of *P.vivax* infection (8.76%)occurred in 2018 among migrant population in Gunung Mas district, while *P.falciparum* infection was found in 2017contributed by both population. The decreased number of cases was shown by very low number of cases among migrant population in almost every year. The observed significant decrease in malaria incidences indicated the success and effective implementation of the malaria control programs at the sub-district level.

**Conclusion:**

To minimize malaria cases among gold miners, mosquito repellent, prophylactic administration of antimalarial drugs and enough enlightenment should be considered before and during their activities. From that standpoint, the effective collaboration between health officers and environmental authorities is recommended to control, prevent and eliminate malaria in these areas.

## Introduction

Parasite-based malaria diagnostic tests either by microscopic examination or RDT is recommended by WHO for all patients suspected of malaria before medication is provided. Microscopy examination on Giemsa-stained blood smears was approved to be the gold standard diagnostic tool for detecting and identifying *Plasmodium* species that caused infection (Mathison *et al*, 2017. After India, Indonesia is the second contributor to the total number of global malaria cases (WHO, 2020). However, the Indonesian ministry of health reported that many districts were certified as malaria –free and the national annual parasite incidence (API) decreased from 1.96 in 2010 to 0.87 in 2020. This shows a significant progress and smooth implementation of malaria control, prevention and elimination programs (Kemenkes RI, 2021).

The province of Central Kalimantan is one of malaria endemic areas that consisted of 13 districts and a capital city. Two of the three districts that have not certified for malaria elimination are Kapuas and Gunung Mas. Palangka Raya City is the capital of this province. This city has been certified malaria free in 2018. Majority of the population in these districts rely on rain-fed agriculture, oil palm plantation and mining. The fact that traditional gold mining practices are increasing in this province (BPS Prov Kalteng, 2017). The big number of migrants from outside these districts and island came to settle and actively involved in mining activities (Lestarisa *et al.*, 2022).

Based on microscopy slides and RDT cartridges collected from both local and migrant populations, the trend of malaria cases in Kapuas, Gunung Mas Districts and capital city of Palangka Raya of Central Kalimantan Province is reported herein to find out the contribution of both local and migrant populations to the malaria cases in the studied areas.

## Materials and Methods

### Description of location

Kapuas and Gunung Mas are the neighboring districts where Gunung Mas district with other two districts (Murung Raya and Barito Utara) border Kapuas district to the North, Java Sea and Barito Kuala district to the South, Pulang Pisau district to the West, Barito Selatan and Barito Kuala districts to the East (RPIJM Kabupaten Kapuas, 2021).Gunung Mas district is bordered by Kapuas District to the East, Katingan district to the West, Murung Raya district to the North, and Pulang Pisau district and Palangka Raya city to the South. The Kapuas District is located on the equator between 0° 8’ 48 “to 3° 27’ 00” South latitude and 113° 2’ 35 “to 114°44’00”. The local population of Kalimantan (formerly was called Borneo) Island is called Dayak (Sada *et al*, 2019). In this study, the migrant population is categorized as non-Dayak people who came from other cities, other provinces and even other islands who have lived in these districts for more than three months (Lestarisa *et al*, 2022).

### Samples collection and ethics

The microcopy and RDT specimens were collected from health centers, Puskesmas Danau Rawah and Puskesmas Kampuri as well as the Puskesmas Bukit Hindu. The two first health centers represented the hypoendemic areas of malaria in Mantangai Sub district of Kapuas District and Mihing Raya Sub district of Gunung Mas District, while the third health center represented the malaria free area in Palangka Raya City. The samples and demographic data of the patients who seek medication to these health centers were collected from 2017 to 2020. The microscopy samples have been examined by trusted analysts in those health centers. The samples were transferred to our laboratory by package delivery service in 2021. The data were then separated into local and migrant populations. The ethical approval of this study was obtained from the Health Research Ethical Clearance Commission of the Faculty of Medicine, Universitas Airlangga, Surabaya, Indonesia with ethical clearance certificate number10/EC/KEPK/FKUA/2021 dated on January 18, 2021.

## Results

### Trend of malaria cases

**Figure 1:**
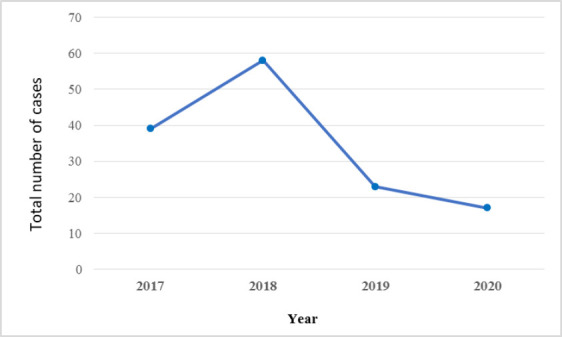
Trend of malaria cases in three hypoendemic areas of malaria in Central Kalimantan Province during 2017-2020.

**Figure 2:**
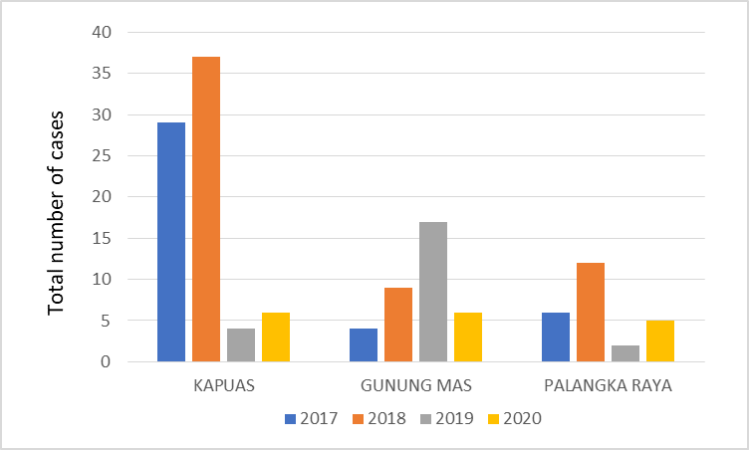
The annual trend of malaria cases in Kapuas District, Gunung Mas District, and Palangka Raya City during 2017-2020 based on microscopy and RDT examinations.

Total number of 140 samples consisting of 75 malaria Giemsa-stained blood smears and 65 RDT cartridges were tested and the results disclosed that only 3(4%) blood smears were negative and 72 (96%) blood smears were positive. Of all collected samples, 2.14 % were negative and 137 (97.85%) samples turned out positive. Of all the positive samples, 39 (27.86%) were from 2017, 58 (41.43%) cases from 2018, 23 (16.43%) cases from 2019, and 17 (12.14%) cases from 2020. The number of cases increased in 2018, however decreased sharply in 2019 and 2020 as shown by the findings presented on Figure 1. The annual trends of malaria cases in every study area is presented in Figure 2. The highest number of malaria cases was found in Kapuas District in 2017 and 2018, but they gradually decreased in 2019 and 2020 compared with the cases found in Gunung Mas district in 2019 and Palangka Raya city in 2018.

### Contribution of local and migrant population

**Figure 3:**
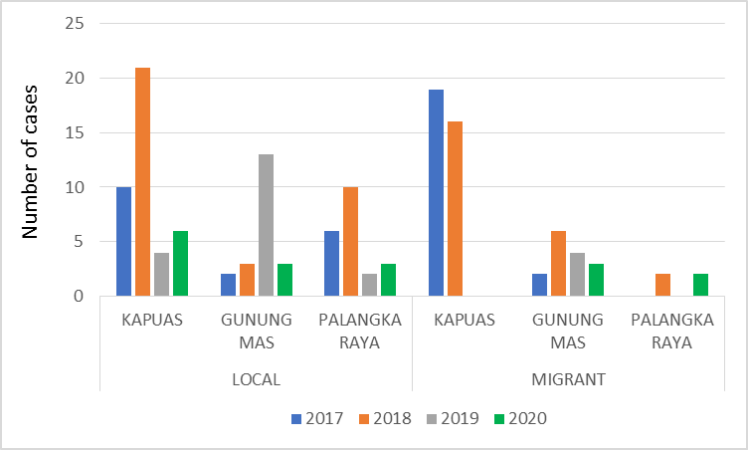
The yearly trend of malaria cases in three studied areas during 2017-2020 in local and migrant populations

The number of malaria cases was classified into local and migrant according to the origins of participants to find out the contribution of each population (Figure 3). The migrant population contributed greatly to the number of malaria cases in Kapuas District during 2017 and 2018, as well as the contribution of the local population in this district. The decline in the number of cases during 2019 and 2020 in all studied areas was shown by the contribution of both migrant and local populations, as shown by the decreasing number of cases in Gunung Mas and Palangka Raya City during those years. There was an increase in malaria cases in Gunung Mas district in 2019 and Palangka Raya City in 2018, but the contribution was low to the overall increase of cases.

### Trend of malaria based on *Plasmodium* species

The trend of malaria cases based on *Plasmodium* species in three studied areas were evaluated. In general, the highest number of malaria cases occurred in Kapuas District. Based on the species, the findings revealed the highest number of *P. vivax* infection (8.76%) occurred in 2017 contributed by migrant population in this district, and then followed by local population which was 7.30% in 2018. The highest number of *P. falciparum* infection was found in 2018 contributed by both local and migrant population (8.03%) in Kapuas District. Although the number of malaria cases decreased during 2019, however the highest number of *P. falciparum* infection was found during this year (5.84%) contributed by local population in Gunung Mas District. Further, the sharp decreased of malaria cases among migrant population showed by only one case of *P. falciparum* infection was found in Gunung Mas district in 2017, 2018 and 2020 and one case of *P. vivax* infection in 2017 and 2019. The same number of cases of *P. vivax* was also reported in Palangka Raya city in 2020. One case of *P. malariae* was found in Kapuas district in 2019 and one case of mixed infection was seen in Palangka Raya city in 2020. *P. falciparum* was found every year among local population in Kapuas district as well as among migrant population in Gunung Mas district. The *P. vivax* infection was found every year among local population in Kapuas District and Palangka Raya city.

### Trend of malaria cases based on demographic data of patients

The demographic characteristics of the enrolled patients are gender, age groups and occupations of both local and migrant patients. The majority of enrolled patients were males whereby 55% were local and 36.49 % were migrants (Figure 5). All subjects aged between 15-65 years old, the biggest number of study subjects were in age range of 15-25 years old (50.36%) (Table 1). Of total 137 samples, 103 (75.18%) were gold miners, consisted of 57 (55.33%) were from local population, while 46 (45.54%) were from migrant population. All female patients were housewives and the other occupations were policemen, government and private employees (Figure 6).

**Figure 4:**
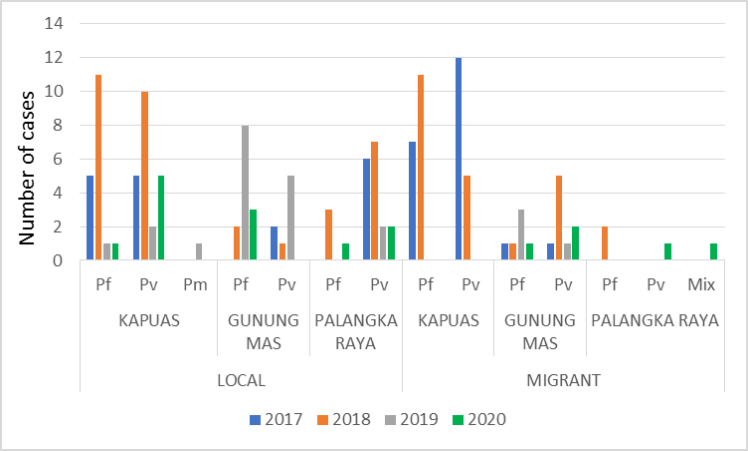
Trend of malaria cases based on the *Plasmodium* species in three studied areas in local and migrant populations. Pf, *P. falciparum*; Pv, *P. vivax*; Pm, *P. malariae*; Mix, mix infection with Pf and Pv.

**Figure 5:**
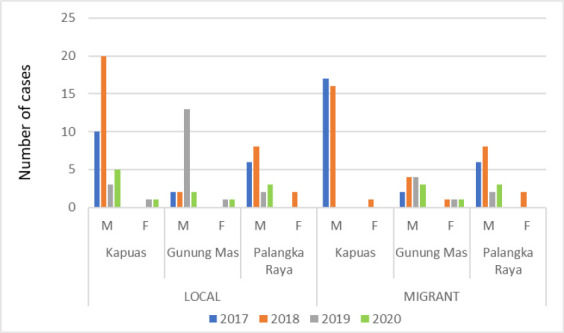
The yearly Trend of malaria cases by gender in local and migrant populations. M, male; F, female

**Table 1:** Trend of malaria patients in studied areas based on the age group

Age (years)	Number of cases (%)				

	2017	2018	2019	2020	TOTAL
15-25	18 (13.14)	33(24.09)	10(7.30)	8(5.84)	69(50.36)
26-35	11(8.03)	11(8.03)	10(7.30)	5(38.46)	37(27.00)
36-45	8(5.84)	13(9.49)	2(1.46)	5(38.46)	28(20.44)
46-55	1(0.73)	1(0.73)	0	0	2(1.46)
56-65	0	1(0.73)	0	0	1(0.73)

TOTAL	38(27.74)	59(43.07)	22(16.06)	18(13.14)	137(100)

**Figure 6:**
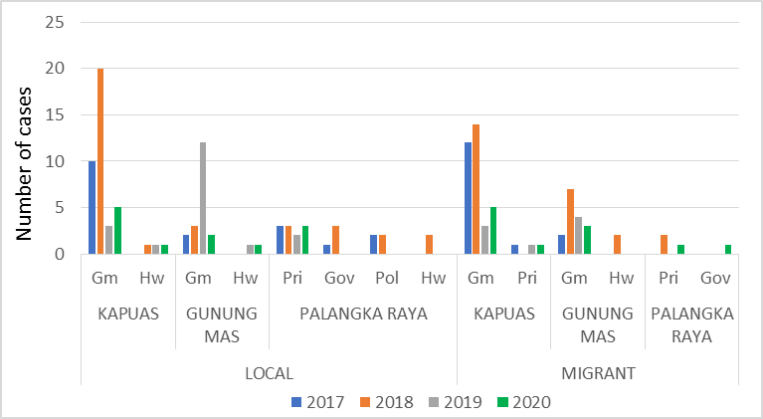
Trend of malaria cases based on the patients’ occupation in local and migrant populations. Gm, goldminer; Hw, housewife; Pri, private company employee, Gov, government employee; Pol, policeman

## Discussion

The malaria elimination target in Indonesia is increasing every year. The trend of malaria in three studied areas showed a decrease year by year and this indicated a success of malaria control program at the sub district level. However, the presence of indigenous malaria cases (Kemenkes RI, 2021), imported malaria (Arwati *et al*, 2019) and submicroscopic malaria (Arwati *et al*, 2018) are some of the challenges in malaria elimination in the hyperendemic areas of malaria.

Kapuas and Gunung Mas districts remained uncertified for malaria elimination due to the presence of indigenous cases while malaria cases found in Palangka Raya city were imported cases. This city has been freed from malaria since 2018 and there was no indigenous malaria case. Published data on the malaria cases in this province is lacked. The statements of the head of Palangka Raya city Health Office reported by the website of local newspaper stated that all malaria cases found in this city were imported cases brought by the local residents and migrants returned from travelling or by migrants who came to visit the city. The public should remain vigilant against the transmission of malaria because *Anopheles* mosquitoes are still existing in this city (Damara, 2021). The occupations of the patients in this city consisted of government and private employees, policemen and housewives, but no gold miner (Figure 5). Interestingly, most occupation of the patients in Kapuas and Gunung Mas districts was gold miner. All of them were male, and mostly were in age range of 15-25 years old (50.36%). This age of group is the productive age. The poverty and difficulties in finding the job forced them to work as illegal gold miners. Gold mining has become an economic necessity for the living in misery, despite they have to put their health at risk (Bose-O’Reilly *et al*, 2010).

The presence of gold mines in tropical countries often caused the increased of malaria cases. In Indonesia, malaria in gold miners have been found in Landak District, West Kalimantan Province (Salim *et al*, 2012) and North Sulawesi Province in Sulawesi Island (Filho *et al*, 2001). A study on the health assessment of artisanal gold miners in Central Kalimantan and North Sulawesi Provinces reported that malaria was one of infectious diseases that caused the morbidity and mortality in both areas (Bose-O’Reilly *et al*, 2010). Miners go and return to and from the gold mines through the forest, where contact with the *Anopheles* mosquito is unavoidable (Indriyati *et al*, 2016). A large correlation between gold mining and malaria can be explained that, gold miners often leave the excavations used to search for gold, and when filled with rainwater become new ponds, and these stagnant water become breeding places of the *Anopheles* mosquito (Bariyah *et al*, 2018, Rozo *et al*,). Besides, the miners came from different areas of malaria endemicity with different immune status and different susceptibility to malaria infection. Malaria in gold mining areas have also been reported from Colombia (Castellanos *et al*, 2016), Guiana and the Amazon (Douine *et al*, 2020), and Brazil (de Andrade *et al*, 2020).

Illegal gold mining is often found in the studied areas. On one hand, gold mining activities can increase family income, however on the other hand illegal gold mining is a hassle for the government because there is no legal permission to operate, it damages the environment, and contaminates the environment with mercury after being used to extract gold, and also increases the number of malaria cases. In fact, illegal gold mining is difficult to eradicate, therefore to decrease malaria among gold miners, alternatively the mosquito repellent should be used during their work, antimalarial prophylactic drugs should be taken before and during their work. An important enlightenment should be given including the dangers of malaria, the prevention, transmission, control and elimination of the disease by health officers in collaboration with the environmental authorities.

## Conclusion

The trend of malaria cases in three studied areas in East Kalimantan Province, Indonesia, indicated the successful of malaria control program at sub-district level. The uncertified in malaria elimination in Kapuas and Gunung Mas districts was due to the indigenous malaria cases contributed by gold miners either from local residents or migrant population. An enlightenment education is needed for the better environment and health relates to artisanal and small-scale gold mining in the studied areas.

### Conflict of interest

The authors declare that there is no conflict of interest associated with this study.

**List of Abbreviations:**
RDT: Rapid Diagnostic Test;
WHO: World Health Organization;
Pf: *Plasmodium falciparum*;
Pv: *Plasmodium vivax*;
Pm: *Plasmodium malariae*;
F: female;
M: male;
Gm: goldminer;
Hw: housewife;
Pri: private company employee,
Gov: government employee;
Pol: policeman
